# Case Report: Buprenorphine-precipitated fentanyl withdrawal treated with high-dose buprenorphine

**DOI:** 10.12688/f1000research.120821.1

**Published:** 2022-05-03

**Authors:** Nicholas L. Bormann, Antony Gout, Vicki Kijewski, Alison Lynch

**Affiliations:** 1 Psychiatry, University of Iowa Hospitals and Clinics, Iowa City, IA, 52242, USA; 2 Internal Medicine, University of Iowa Hospitals and Clinics, Iowa City, IA, 52242, USA; 3 Family Medicine, University of Iowa Hospitals and Clinics, Iowa City, IA, 52242, USA

**Keywords:** Buprenorphine, fentanyl, Opioid-Related Disorders, case report

## Abstract

**Background:** Buprenorphine, a partial agonist of the mu-opioid receptor, is an increasingly prescribed medication for maintenance treatment of opioid use disorder. When this medication is taken in the context of active opioid use, precipitated withdrawal can occur, leading to acute onset of opioid withdrawal symptoms. Fentanyl complicates use of buprenorphine, as it slowly releases from body stores and can lead to higher risk of precipitated withdrawal.

**Objectives:** Describe the successful management of buprenorphine precipitated opioid withdrawal from fentanyl with high doses of buprenorphine. We seek to highlight how no adverse effects occurred in this patient and illustrate his stable transition to outpatient treatment.

**Case report:** We present the case of a patient with severe opioid use disorder who presented in moderately severe opioid withdrawal after taking non-prescribed buprenorphine-naloxone which precipitated opioid withdrawal from daily fentanyl use. He was treated with high doses of buprenorphine, 148 mg over the first 48 hours, averaging 63 mg per day over four days. The patient reported rapid improvement in withdrawal symptoms without noted side effects and was able to successfully taper to 16 mg twice daily by discharge.

**Conclusions:** This case demonstrates the safety and effectiveness of buprenorphine at high doses for treatment of precipitated withdrawal. While other options include symptomatic withdrawal management, initiating methadone or less researched options like ketamine, utilizing buprenorphine can preserve or re-establish confidence in this life-saving medication. This case also increases the previously documented upper boundary on buprenorphine dosing for withdrawal and should provide additional confidence in its use.

## Introduction

Buprenorphine, a partial mu-opioid receptor agonist, has become the most prescribed treatment for opioid use disorder (OUD). ^1^ With greater availability, non-prescribed use has also increased. ^2^ When buprenorphine is taken after recent use of a full-agonist opioid, buprenorphine displaces the lower affinity molecule, causing a precipitated withdrawal. ^3^ This rapid onset of opioid withdrawal symptoms including bone and muscle pain, diarrhea, insomnia, dysphoria, and anxiety, causes significant patient discomfort.

The rise of synthetic opioids such as fentanyl has complicated the treatment of precipitated withdrawal. Fentanyl is more potent than heroin, and has high lipophilicity leading to rapid uptake into body tissues and subsequent slow release. ^4^^,^^5^ While use of ketamine has been suggested, ^6^ conventional wisdom has been to utilize additional buprenorphine. ^7^ Recent cases in the literature have shown safety and effectiveness of up to 40 mg of buprenorphine early in the withdrawal period. ^7^^–^^11^ Herein, we describe the case of a patient who required 148 mg of buprenorphine over 48 hours for successful treatment of buprenorphine-precipitated withdrawal from fentanyl. Consent was obtained from the patient for his case to be used in the academic literature and is available upon request. As such, the University of Iowa Institutional Review Board has deemed it exempt.

## Case description

A 21-year-old partnered, unemployed Caucasian male with no known past medical history, a psychiatric history of attention deficit hyperactivity disorder and unspecified anxiety, with a pertinent family history of an opioid use disorder in his brother and both his mother and maternal aunt having unspecified addiction to pills per his father, who lived in an apartment with a roommate however was being evicted due to late rent payments, presented with his father to our medication for addiction treatment (MAT) walk-in clinic located at a primary care outreach clinic for assistance discontinuing daily fentanyl use. Initial opioid exposure was through purchasing prescription opioids for six months, before transitioning to use of fentanyl after he had purchased it unknowingly. He endorsed daily fentanyl use of an unknown amount for six months, with an escalation in patient-estimated amount over the preceding two months. His typical method of use was insufflation or vaping. He had attempted to stop multiple times by tapering use with the goal of abstinence, however, was unsuccessful after occurrence of withdrawal symptoms led to eventual return to daily use of the previous amount. Other substance use consisted of non-prescribed alprazolam 1 mg daily that he had started taking in the previous weeks for anxiety symptoms. He denied other active substance use.

After making plans to present to the MAT clinic, he abstained from using fentanyl to prepare for buprenorphine induction. His father drove him to the MAT clinic sixteen hours after last fentanyl use. While traveling to the clinic, he took non-prescribed buprenorphine-naloxone 8-2 mg, which immediately precipitated withdrawal. On initial evaluation in clinic, he had a clinical opioid withdrawal scale ^12^ (COWS) score of 27, with diffuse pain, nausea, emesis, diarrhea, rhinorrhea, chills, yawning, anxiety, and restlessness. He also was intermittently agitated and having visual hallucinations. Due to lack of readily available medications in the outreach clinic, he was taken to the emergency department (ED) at the main hospital.

At the ED, he was given buprenorphine monoproduct (referred to herein as buprenorphine) 8 mg, which lowered COWS to 19 and provided approximately 45 minutes of relief. Screening labs in the ED consisted of a complete metabolic panel, complete blood count with differential, urinalysis, blood alcohol level, acetaminophen drug level, urine drug screen and electrocardiogram (ECG). This standard screening panel was largely within normal limits, with positive findings of presumptive positive for urine benzodiazepines, a minor elevation in neutrophils (count of 7,850 with normal range of 2,188 – 7,800/mm ^3^), and ECG QTc interval of 463 millisecond (normal less than 430 millisecond). He received an additional 8 mg of buprenorphine 3 hours later which lowered COWS to 9. He subsequently was administered doses of 4 mg three times over the next 8 hours, which he did not feel provided as much relief as higher doses. This was increased to 16 mg doses of buprenorphine, which he tolerated without significant changes in vital signs, and provided symptomatic relief for 2 hours at a time. Medication administration record for buprenorphine can be seen in Table 1.

**Table 1.** Buprenorphine administration timetable.

Hospital Day	Time	Hours post t _0_	Buprenorphine, mg	Buprenorphine 24-hour dose, mg
1	16:27	2.0	8	68
	19:27	5.0	8	
	22:10	7.7	4	
2	1:03	10.6	4	
	4:03	13.6	4	
	6:19	15.8	8	
	8:25	17.9	16	
	11:21	20.9	16	
	15:36	25.1	16	80
	19:02	28.5	16	
	22:26	31.9	16	
3	0:48	34.3	16	
	8:38	42.1	16	
	15:15	48.8	16	56
	21:59	55.5	16	
4	8:58	66.5	16	
	11:06	68.6	8	
	16:12	73.7	8	48
	20:24	77.9	8	
	21:02	78.5	16	
5	8:44	90.2	16	
	22:24	103.9	8	24
6	8:48	114.3	16	
	22:13	127.7	16	32
7	9:35	139.1	16	

t _0_ = 14:30; time at which patient took initial buprenorphine-naloxone 8-2 mg dose that precipitated his withdrawal.

Each horizontal bolded line signifies separation of a 24-hour period, with initial reference time of t _0_. The time column indicates hospital clock time. The values in far-right column are for each subsequent 24-hour period after the initial precipitated withdrawal.

Over the first 24 hours, he received 68 mg of buprenorphine and was routinely assessed by nursing. He became physically restless with increasing anxiety, however these improved with repeat dosing of buprenorphine. He was seen by a psychiatrist in the ED, who recommended admission to the medical-psychiatry unit for on-going management of the high-doses of buprenorphine. The patient’s goal was long-term abstinence from fentanyl use, and continued treatment with buprenorphine was felt to be the most direct method to accomplish that.

Over the second 24-hour period from initial precipitation of withdrawal, he received an additional 80 mg of buprenorphine in 16 mg doses. His respiratory rate measured consistently between 16 and 18 breaths per minute (normal is typically considered 12 to 16) during this time. His daily buprenorphine dose requirement peaked on day two, with the goal for discharge of 16 mg twice daily, the maximum daily amount his insurance would cover. He tolerated this reduction over three days without exacerbation of symptoms or cravings.

The COWS was scheduled every 4 hours, and his scoring remained low with continued treatment. In addition to buprenorphine, he received gabapentin 300 mg thrice daily titrated to 600 mg thrice daily for physical discomfort and anxiety. His anxiety was worse earlier in his course, and he received lorazepam while on the unit, averaging a daily dose of 2 mg by mouth. He was discharged on 1 mg daily of clonazepam. He is now seen in the MAT outpatient clinic and has a severe opioid use disorder in early remission on buprenorphine-naloxone 24-6 mg once daily. His clonazepam use has been tapered, now taking less than 0.5 mg daily. He reports increased stability in his life, and relayed appreciation for the care and assistance he received in transitioning to MAT. He is followed by the senior author, who also saw him at both the walk-in clinic and main hospital. VK was the attending on the inpatient unit during his stay.

## Discussion

This case builds upon existing literature by extending the upper extreme of known buprenorphine dosing for treatment of buprenorphine precipitated withdrawal. Previous case reports have used between 16 and 40 mg daily. ^7^^–^^11^ In this case the range was doubled without significant adverse effect. A separate trial showed evidence for tolerability of one-time dosing of up to 96 mg of buprenorphine with goal of craving reduction. ^13^ This however, to our knowledge, is the first report of a patient tolerating repeated days of high dose buprenorphine, averaging 63 mg per day over the first four days, with maximum 24-hour dose of 80 mg.

The US Food and & Drug Administration (FDA) has approved use of buprenorphine up to 32 mg daily. At this dose, the mu-opioid receptor nears saturation. ^14^ Buprenorphine’s duration of action however is only 6–8 hours, ^15^ which may partially explain the effectiveness of repeated high doses in this and other cases. Further research is needed in this area, such as incorporation of imaging techniques to quantify changes in receptor occupancy with re-dosing of buprenorphine.

Opioid withdrawal is a strong negative reinforcer for patients, and the fear of withdrawal may prompt behavior changes intended to avoid such misery. ^16^ Withdrawal specifically precipitated by buprenorphine is felt by some to be particularly uncomfortable, due to the abrupt displacement of opioid agonist by the high affinity buprenorphine molecule. ^3^ It is reasonable that an experience with buprenorphine leading to withdrawal would limit one’s willingness to continue taking it or to utilize it for long-term maintenance therapy. ^17^ However, with only three FDA approved medications for treatment of OUD and buprenorphine being the most readily accessible, attempts should be made to reassure the patient, optimize the initial exposure to buprenorphine even if use prompted a visit to the ED, and preserve or reestablish the patient’s confidence that this medication can provide benefit.

Along with administering additional buprenorphine, alternative recommendations for the treatment of acute buprenorphine precipitated withdrawal are discontinuation with symptomatic treatment (such as clonidine, ondansetron, loperamide and gabapentin) or utilization of full-opioid agonist such as methadone. ^7^ In the moment, discontinuing the medication that caused acute withdrawal while providing symptomatic treatment may feel like the safest option, but this strategy misses an opportunity to initiate a potentially life-saving medication. Obtaining a start date for methadone maintenance therapy at an opioid treatment program out of the ED is also a large barrier to care and unrealistic for most parts of the United States. Ketamine has also been suggested, specifically in the ED. ^6^ A noncompetitive N-methyl-D-aspartate (NMDA) receptor antagonist, ketamine has the potential to suppress physiologic symptoms of withdrawal. ^18^ In this patient, continued use of buprenorphine throughout his hospital stay helped the patient develop confidence in the medication and incorporate taking it into his daily routine.

A commonly voiced concern for escalating doses of buprenorphine is respiratory depression. Buprenorphine’s effect on ventilation has been shown to plateau with a ceiling effect, unlike fentanyl, which can lead to apnea with increasing dose. ^19^ While this case along with others ^7^^–^^11^ have shown no issues with respiratory depression, it remains a practical concern. Scheduling the COWS can help mitigate this risk by utilizing symptom triggered dosing to inform daily requirement. Contamination of the drug supply, particularly with fentanyl, may also contribute to risk of respiratory depression. The occurrence of hallucinations in the case may be due to opioid withdrawal, which is a documented but less frequent symptom, ^20^ or from a contaminant.

To conclude, this case provides additional evidence for the tolerability of high-dose buprenorphine and how the medication can be successfully tapered to a safe outpatient dose by discharge. There were no identifiable side effects to this total dosing of buprenorphine. As these doses exceeded the FDA approved limit, the use we describe is off label. This report builds upon existing literature and should provide additional confidence for providers in the emergency setting to opt for treatment of precipitated withdrawal with high-dose buprenorphine.

## Data availability

All data underlying the results are available as part of the article and no additional source data are required.

## Reporting guidelines

Open Science Framework: CARE checklist for ‘Buprenorphine-precipitated fentanyl withdrawal treated with high-dose buprenorphine: a case report’. http://doi.org/10.17605/OSF.IO/9M468. ^21^

Data are available under the terms of the Creative Commons Zero “No rights reserved” data waiver (CC0 1.0 Public domain dedication).

## Consent

Written informed consent for publication of their clinical details was obtained from the patient.
