# Supplementary material for: Case Report: Buprenorphine-precipitated fentanyl withdrawal treated with high-dose buprenorphine
Source: F1000Res. 2023 Aug 10;11:487. Originally published 2022 May 3. [Version 2] doi: 10.12688/f1000research.120821.2 (PMC10521070; doi:10.12688/f1000research.120821.2)
Supplement: Peer review history

# Reviewer report: Reviewer response for version 2

**DOI:** 10.5256/f1000research.154251.r195439

**Published:** 2023-09-07

**Authors:** Neil B. Varshneya

**Affiliations:** 1 Center for Drug Evaluation and Research, Food and Drug Administration, United States Department of Health and Human Services, Silver Spring, MD, USA

The authors satisfactorily addressed my comments.

Are enough details provided of any physical examination and diagnostic tests, treatment given and outcomes?

Yes

Is the case presented with sufficient detail to be useful for other practitioners?

Yes

Is sufficient discussion included of the importance of the findings and their relevance to future understanding of disease processes, diagnosis or treatment?

Yes

Is the background of the case’s history and progression described in sufficient detail?

Yes

Reviewer Expertise:

Pharmacology (fentanyl)

I confirm that I have read this submission and believe that I have an appropriate level of expertise to confirm that it is of an acceptable scientific standard.

# Reviewer report: Reviewer response for version 1

**DOI:** 10.5256/f1000research.132683.r189490

**Published:** 2023-08-03

**Authors:** Ethan O Bryson

**Affiliations:** 1 Icahn School of Medicine at Mount Sinai, New York, USA

This case report describes the use of high-dose buprenorphine in the treatment of buprenorphine-precipitated fentanyl withdrawal. Given the considerable increase in fentanyl-adulterated opioids and non-opioids as well as an increase in persons with opioid use disorder specifically seeking out fentanyl this report is timely and appropriate.

 There are some grammatical errors that need to be addressed prior to indexing, however, specifically:

 "While use of ketamine has been suggested,6 conventional wisdom has been to utilize additional buprenorphine.7" Please specify that this is for withdrawal treatment to make this a complete thought.

 "As such, the University of Iowa Institutional Review Board has deemed it exempt." please specify what is exempt from what.

 "A 21-year-old partnered, unemployed Caucasian male with no known past medical history, a psychiatric history of attention deficit hyperactivity disorder and unspecified anxiety, with a pertinent family history of an opioid use disorder in his brother and both his mother and maternal aunt having unspecified addiction to pills per his father, who lived in an apartment with a roommate however was being evicted due to late rent payments, presented with his father to our medication for addiction treatment (MAT) walk-in clinic located at a primary care outreach clinic for assistance. discontinuing daily fentanyl use."

 This sentence is too long, and the reader may wonder who (the father? the aunt? or the patient) was late on their rent. Only include details like this if they are relevant. Also, please add "with opioid use disorder" in the patients' history or, if not previously diagnosed, something to that effect as this is more relevant than the other information which is included.

Are enough details provided of any physical examination and diagnostic tests, treatment given and outcomes?

Yes

Is the case presented with sufficient detail to be useful for other practitioners?

Yes

Is sufficient discussion included of the importance of the findings and their relevance to future understanding of disease processes, diagnosis or treatment?

Yes

Is the background of the case’s history and progression described in sufficient detail?

Yes

Reviewer Expertise:

Addiction, Anesthesiology, Electroconvulsive therapy

I confirm that I have read this submission and believe that I have an appropriate level of expertise to confirm that it is of an acceptable scientific standard.

# Reviewer report: Reviewer response for version 1

**DOI:** 10.5256/f1000research.132683.r184916

**Published:** 2023-08-03

**Authors:** Neil B. Varshneya

**Affiliations:** 1 Center for Drug Evaluation and Research, Food and Drug Administration, United States Department of Health and Human Services, Silver Spring, MD, USA

Bormann et al. describe the successful management of buprenorphine-precipitated fentanyl withdrawal with high-dose buprenorphine and provide valuable insights into the management of opioid use disorder and the challenges associated with fentanyl exposure. This case report underscores the importance of individualized medication management in patients with opioid use disorder. The patient in this case had a history of opioid use disorder, including repeated fentanyl self-administration (with daily fentanyl use and dose escalation for 6 months), and developed severe withdrawal symptoms following non-medical, unsupervised self-treatment with buprenorphine/naloxone. The patient was subsequently managed in a medical setting with high doses of buprenorphine, which provided symptomatic relief in two hours intervals. The patient was eventually stabilized on a lower dose of buprenorphine and was discharged without exacerbation of symptoms or cravings. The report highlights the need for careful monitoring and tailored treatment plans for patients with opioid use disorder, especially in cases of fentanyl exposure. The report also underscores the importance of proper medication management for the acute onset of opioid withdrawal symptoms.

 Overall, this is a well-written an interesting manuscript and is timely given the role of fentanyl and its analogs in the ongoing opioid crisis. In general, the data are clearly presented. However, there are a few minor issues that deserve attention:

  

1. The authors write: “When buprenorphine is taken after recent use of a full-agonist opioid, buprenorphine displaces the lower affinity molecule, causing a precipitated withdrawal.” I think there are instances in which buprenorphine, with high enough doses, would still precipitate withdrawal by reducing receptor activation when competing with mu-opioid receptor ligands of higher affinity and efficacy. You should consider rewriting this sentence as follows: “Buprenorphine can precipitate withdrawal by reducing receptor activation through competitive displacement of higher efficacy mu-opioid receptor ligands”. Thank you for citing my work here.
2. The authors write: “Further research is needed in this area, such as incorporation of imaging techniques to quantify changes in receptor occupancy with re-dosing of buprenorphine.” You should consider including additional research strategies from the following article and cite it here:
  Volkow, N. D., & Blanco, C. (2023, Jun 1). Fentanyl and Other Opioid Use Disorders: Treatment and Research Needs. Am J Psychiatry, 180(6), 410-417. https://doi.org/10.1176/appi.ajp.20230273
3. The authors write: “However, with only three FDA approved medications for treatment of OUD and buprenorphine being the most readily accessible, attempts should be made to reassure the patient, optimize the initial exposure to buprenorphine even if use prompted a visit to the ED, and preserve or reestablish the patient’s confidence that this medication can provide benefit.” Please list the three FDA approved medications in parentheses following “only three FDA approved medications for treatment of OUD”.
4. The authors write: “A commonly voiced concern for escalating doses of buprenorphine is respiratory depression. Buprenorphine’s effect on ventilation has been shown to plateau with a ceiling effect, unlike fentanyl, which can lead to apnea with increasing dose.” You might consider citing the following study showing demonstrating the respiratory-depressant ceiling of buprenorphine vs fentanyl in animal models:
  Varshneya, N. B., Hassanien, S. H., Holt, M. C., Stevens, D. L., Layle, N. K., Bassman, J. R., Iula, D. M., & Beardsley, P. M. (2022, Jan). Respiratory depressant effects of fentanyl analogs are opioid receptor-mediated. Biochem Pharmacol, 195, 114805. https://doi.org/10.1016/j.bcp.2021.114805
5. The authors write: “This report builds upon existing literature and should provide additional confidence for providers in the emergency setting to opt for treatment of precipitated withdrawal with high-dose buprenorphine.” I wouldn’t say that N = 1 exactly inspires confidence, so I suggest using different language here to state that further research is needed.

Are enough details provided of any physical examination and diagnostic tests, treatment given and outcomes?

Yes

Is the case presented with sufficient detail to be useful for other practitioners?

Yes

Is sufficient discussion included of the importance of the findings and their relevance to future understanding of disease processes, diagnosis or treatment?

Yes

Is the background of the case’s history and progression described in sufficient detail?

Yes

Reviewer Expertise:

Pharmacology (fentanyl)

I confirm that I have read this submission and believe that I have an appropriate level of expertise to confirm that it is of an acceptable scientific standard, however I have significant reservations, as outlined above.

## References

[1] : Fentanyl and Other Opioid Use Disorders: Treatment and Research Needs. *Am J Psychiatry* .2023;180(6) : 10.1176/appi.ajp.20230273 410-417 10.1176/appi.ajp.20230273 37259512

[2] : Respiratory depressant effects of fentanyl analogs are opioid receptor-mediated. *Biochemical Pharmacology* .2022;195: 10.1016/j.bcp.2021.114805 10.1016/j.bcp.2021.114805 PMC902237134673011
